# Effect of SGLT2 inhibitors on mean platelet volume in heart failure with reduced ejection fraction: a real-world analysis independent of diuretic therapy

**DOI:** 10.1007/s11739-026-04398-8

**Published:** 2026-06-23

**Authors:** Hüseyin Orta, Cihan Aydin, Aykut Demirkiran, Ferhat Perk, Aydin Akyuz

**Affiliations:** Department of Cardiology, Faculty of Medicine, Tekirdag Namik Kemal University, 59030 Tekirdag, Türkiye

**Keywords:** Heart failure, Sodium-glucose co-transporter 2 inhibitors, Mean platelet volume

## Abstract

Sodium-glucose co-transporter 2 (SGLT2) inhibitors have emerged as cornerstone therapies for heart failure with reduced ejection fraction (HFrEF), owing to their cardioprotective and hematologic effects. While their impact on hemoglobin and hematocrit levels is increasingly recognized, the influence of SGLT2 inhibitors on mean platelet volume (MPV), a surrogate marker of platelet activation and cardiovascular risk, remains underexplored in HFrEF patients. While SGLT2 inhibitors’ effects on MPV have been studied in DM, this is the first study examining MPV changes specifically in HFrEF patients with adjustment for diuretic therapy. This retrospective study included 80 HFrEF patients receiving guideline-directed medical therapy to which SGLT2 inhibitors were subsequently added. Baseline and 6-month follow-up data on hematological and biochemical parameters were collected. Exclusion criteria included active infection, malignancy, advanced renal failure, hematologic disorders, and recent transfusions. MPV and platelet counts were analyzed using standardized protocols and equipment. Following 6 months of SGLT2 inhibitor therapy, MPV values decreased significantly (*p* < 0.05), while platelet counts increased significantly (*p* < 0.05). Although hemoglobin and hematocrit levels showed upward trends, these changes were not statistically significant. No significant correlation was observed between ΔMPV and ΔPLT. Other biochemical markers remained stable throughout the study period. SGLT2 inhibitor therapy was associated with a significant reduction in MPV and an increase in platelet count among patients with HFrEF. These hematological changes may represent an additional mechanism by which SGLT2 inhibitors exert cardiovascular benefit. However, prospective, randomized trials are needed to validate these findings and explore the clinical significance of MPV modulation in heart failure management.

## Introduction

Heart failure (HF) is defined as a clinical syndrome encompassing all structural and/or functional cardiac disorders that impair the heart’s ability to deliver oxygen at a rate commensurate with the metabolic needs of tissues and organs. The classification of HF by the European Society of Cardiology (ESC) is based on left ventricular ejection fraction (EF), disease progression, and symptom severity [1].

The EF-based classification is considered the fundamental framework for guiding medical therapy. Left ventricular EF is calculated by dividing stroke volume (end-diastolic volume minus end-systolic volume) by the end-diastolic volume. Patients with an EF below 40% are classified as having heart failure with reduced EF (HFrEF), those with an EF of 50% or higher as heart failure with preserved EF (HFpEF), and those with an EF between 40 and 49% as heart failure with mildly reduced EF (HFmrEF) [1].

In patients with HFrEF, beta-blockers, angiotensin-converting enzyme inhibitors (ACE-Is), angiotensin II receptor blockers (ARBs), angiotensin receptor-neprilysin inhibitors (ARNIs), mineralocorticoid receptor antagonists (MRAs), and sodium-glucose co-transporter [2] (SGLT2) inhibitors are first-line treatments due to their mortality-reducing effects. SGLT2 inhibitors are noteworthy for reducing hospitalizations related to heart failure (HF) regardless of ejection fraction (EF).

SGLT2 inhibitors lower plasma glucose levels by inhibiting glucose reabsorption in the renal tubules [2]. They also inhibit sodium reabsorption, exerting a natriuretic effect. The resulting osmotic diuresis leads to volume loss and consequently lowers blood pressure. SGLT2 inhibitors contribute further through weight loss and suppression of sympathetic activity [3]. They exert favorable effects on lipid profiles, including modest increases in total cholesterol, LDL cholesterol, and HDL cholesterol, accompanied by reductions in triglyceride levels, with evidence suggesting a shift toward less atherogenic LDL subfractions [4, 5]. By stimulating erythropoietin production, SGLT2 inhibitors increase hematocrit and hemoglobin levels, a crucial benefit for meeting tissue oxygen demands in HF patients [6].

There is a significant correlation between platelet volume and activity [7]. Therefore, increased mean platelet volume (MPV) is considered an independent predictor of vascular events [8–12]. However, the specific effect of SGLT2i on MPV in HFrEF patients, independent of concurrent diuretic therapy, remains unexplored. Given that HFrEF patients routinely receive loop/thiazide diuretics, which may cause hemoconcentration and affect platelet indices, understanding the isolated impact of SGLT2i on MPV in this unique population is clinically relevant for risk stratification and therapeutic optimization.

Several studies have shown that SGLT2 inhibitors may reduce platelet activation, particularly in diabetic populations [13–15].

However, data on the effects of SGLT2 inhibitors on mean platelet volume (MPV) in patients with HFrEF remain limited. This question is particularly relevant because these patients routinely receive loop and/or thiazide diuretics, which may induce hemoconcentration and alter platelet indices, potentially confounding MPV measurements. Evaluating the impact of SGLT2 inhibitors on MPV independent of background diuretic therapy may therefore provide additional insight into their hematological and cardiovascular effects. Accordingly, this study aimed to evaluate the effect of SGLT2 inhibitor therapy on MPV in patients with HFrEF, given the established association between MPV and cardiovascular risk.

## Materials and methods

After reviewing the records of patients who presented to our cardiology outpatient clinic between January 2024 and December 2025, 80 patients diagnosed with heart failure with reduced ejection fraction (HFrEF) were retrospectively included in the study. This was a single-center study conducted at the Department of Cardiology, Tekirdag Namik Kemal University Faculty of Medicine, Türkiye. The study population consisted predominantly of Turkish patients. All eligible patients who met the predefined inclusion and exclusion criteria during the study period were consecutively included. No randomization was performed due to the retrospective design of the study. All patients were already receiving the maximum tolerated doses of guideline-directed medical therapy according to ESC recommendations when SGLT2 inhibitor therapy was added. During the 6-month follow-up period, no changes were made to the patients’ baseline medications or the SGLT2 inhibitor regimen. Biochemical tests and complete blood count data were recorded at baseline and after 6 months. The diagnosis of heart failure was made by the 2021 ESC heart failure guidelines. Coronary artery disease (CAD) was defined as greater than 50% stenosis in the lumen of the epicardial coronary arteries. Peripheral arterial disease (PAD) was defined as an ankle-brachial index value < 0.9. None of the patients had advanced PAD requiring amputation. Cerebrovascular events (CVE) were defined as neurological deficits due to ischemic or hemorrhagic causes. Demographic and clinical data including age, sex, presence of CAD, DM, CVE, atrial fibrillation, hypertension, smoking status, and medications used were recorded. Antithrombotic therapy was recorded. The effects of these agents on platelet indices are well-established; therefore, any observed changes in MPV were interpreted in the context of stable background therapy.

Exclusion criteria were: significant valvular heart disease, morbid obesity (BMI > 40 kg/m^2^), acute infection, patients using anticoagulants for any reason, thyroid and hepatic disorders, a history of malignancy, receipt of erythrocyte or fresh frozen plasma transfusion during the previous 6 months, advanced renal failure, and hematologic disorders (e.g., polycythemia vera and thrombophilia).

The study was approved by the Non-Interventional Clinical Research Ethics Committee of Tekirdag Namik Kemal University Faculty of Medicine and was conducted in accordance with the Declaration of Helsinki.

## Laboratory measurements

Hematological parameters, including red and white blood cell counts, MPV, and platelet count, were analyzed using a Roche Sysmex XT-2000i automated analyzer (Roche Diagnostics, Paris, France), with the same commercial kits employed at both baseline and the 6-month follow-up. Blood samples were collected in 2 mL vacutainer tubes containing dipotassium EDTA (Vacutainer, Becton, Dickinson and Company, Franklin Lakes, New Jersey). Analyses were performed within 30 min according to the biochemistry department’s protocol. Both intra- and inter-assay coefficients of variation were below 5% for all measurements. Fasting antecubital venous blood samples were collected for the measurement of fasting glucose, glycated hemoglobin, urea, creatinine, total cholesterol (TC), triglycerides (TG), HDL-C, LDL-C, and ProBNP. The same vacutainer tubes and anticoagulants were used, and all MPV measurements during data collection were conducted using the same analyzer.

## Statistical analysis

Statistical analysis was performed using SPSS Statistics 18 for Windows (SPSS Inc, Chicago, Illinois). Results were expressed as mean ± standard deviation or as percentages. The Kolmogorov–Smirnov test was used to assess the distribution of continuous variables. Baseline and 6-month follow-up measurements were compared using paired t-tests. Mann–Whitney U test was used to evaluate MPV change in subgroup analysis. The relationship between MPV and platelet count (PLT) was evaluated using Spearman correlation analysis. Changes in hematological and biochemical parameters before and after SGLT2 inhibitor treatment were calculated as deltas (Δ). A p-value of < 0.05 was considered statistically significant.

## Results

The demographic characteristics of the patients are summarized in Table 1. Following SGLT2 inhibitor use, MPV values significantly decreased from 11.4 ± 0.7 fL at baseline to 9.1 ± 0.9 fL at 6 months (*p* < 0.001), indicating a reduction in platelet activation. In contrast, platelet counts significantly increased from 236 ± 74 × 10⁹/L to 256 ± 76 × 10⁹/L (*p* = 0.009). Although hemoglobin (HGB) and hematocrit (HCT) levels increased, these changes did not reach statistical significance (Table 2). No correlation was found between ΔPLT and ΔMPV (*r* =  − 0.1, *p* = 0.363).

**Table 1:** Baseline clinical characteristics of the study population

Variables	Patients (*n* = 80)
Male, *n* (%)	53 (67.1)
Age (years)	64.6 ± 12
Hypertension, *n* (%)	69 (87.3)
Diabetes mellitus, *n* (%)	52 (66.7)
Stroke, n(%)	15 (18.7)
Coronary artery disease, *n* (%)	58 (72.5)
Peripheral artery disease, *n* (%)	20 (25)
Ejection fraction%	34.8 (17–40)
LVEDD (mm)	59.2 (43–70)
LVESD (mm)	45 (32–60)
Medical treatment	
ACE/ARB, *n* (%)	59 (74.7)
Antiagregans, *n* (%)	58 (72.5)
ARNI, *n*(%)	19 (23.7)
Beta -blocker, *n* (%)	74 (92.5)
MRA, *n* (%)	71 (88.7)
Statin, *n* (%)	62 (77.5)
Diuretics, *n* (%)	74 (92.5)
SGLT-2 inhibitors, *n* (%)	
Empagliflozin, *n* (%)	28 (35)
Dapagliflozin, *n* (%)	52 (65)

*HDL-C* high-density lipoprotein cholesterol, *LDL-C* low-density lipoprotein cholesterol, *LVEDD* left ventricle end-diastolic diameter, *LVESD* left ventricular end-systolic diameter, *ACE* angiotensin-converting enzyme inhibitors, *ARB* angiotensin receptor blockers, *ARNI* angiotensin II receptor blocker and a neprilysin inhibitor, *MRA* mineralocorticoid receptor antagonist, *SGLT-2 inhibitors* sodium-glucose transport protein 2 (SGLT2) inhibitors

**Table 2:** Biochemical and hematological measurements of the study population

Biochemical and hematological measurements	Pre-treatment	Post-treatment	*p* value
Hemoglobin (g/dL)	13.7 ± 2.3	14.3 ± 1.7	0.679
Hematocrit	40.6 ± 7.7	41.9 ± 6.4	0.101
Mean corpuscular volume, fL	89 ± 6.5	87 ± 10.8	0.266
Mean platelet volume, fL	11.4 ± 0.7	9.1 ± 0.9	** < 0.001**
Platelet (X10^9^/L)	236 ± 74	256 ± 76	**0.009**
Pro–B-type natriuretic peptide (pg/mL)	6949 ± 926	6178 ± 871	0.546
High sensitivity Troponin I (ng/mL)	172 ± 69	58 ± 10	0.356
Creatinine (mg/dL)	1.05 ± 0.3	1.1 ± 0.3	0.08
High-sensitivity c-reactive protein test (mg/L)	14.1 ± 3	18.4 ± 5	0.437
LDL-C	110 ± 41	102 ± 39	0.276
Dapagliflozin ΔMPV(fL) (min.-max.)	− 2(− 2.8–1.5)		**0.03**
Empagliflozin ΔMPV(fL)(min.-max.)	− 0.6(− 2.4–2)		

*LDL-C*, low-density lipoprotein cholesterol

Bold values indicate statistically significant results (p<0.05)

To evaluate whether the observed increase in platelet count was secondary to hemoconcentration, we analyzed the correlation between changes in hematocrit (ΔHCT) and platelet count (ΔPLT).

Despite numerical increases in hemoglobin (13.7 ± 2.3 to 14.3 ± 1.7 g/dL, *p* = 0.679) and hematocrit (40.6 ± 7.7 to 41.9 ± 6.4%, *p* = 0.101), these changes did not reach statistical significance. More importantly, no significant correlation was found between ΔHCT and ΔPLT (*r* =  − 0.08, *p* = 0.465), suggesting that the platelet count increase was not merely a consequence of hemoconcentration. Furthermore, the discordant pattern of decreased MPV and increased platelet count suggests that mechanisms other than simple hemoconcentration may have contributed to the observed changes.

**Fig. 1:**
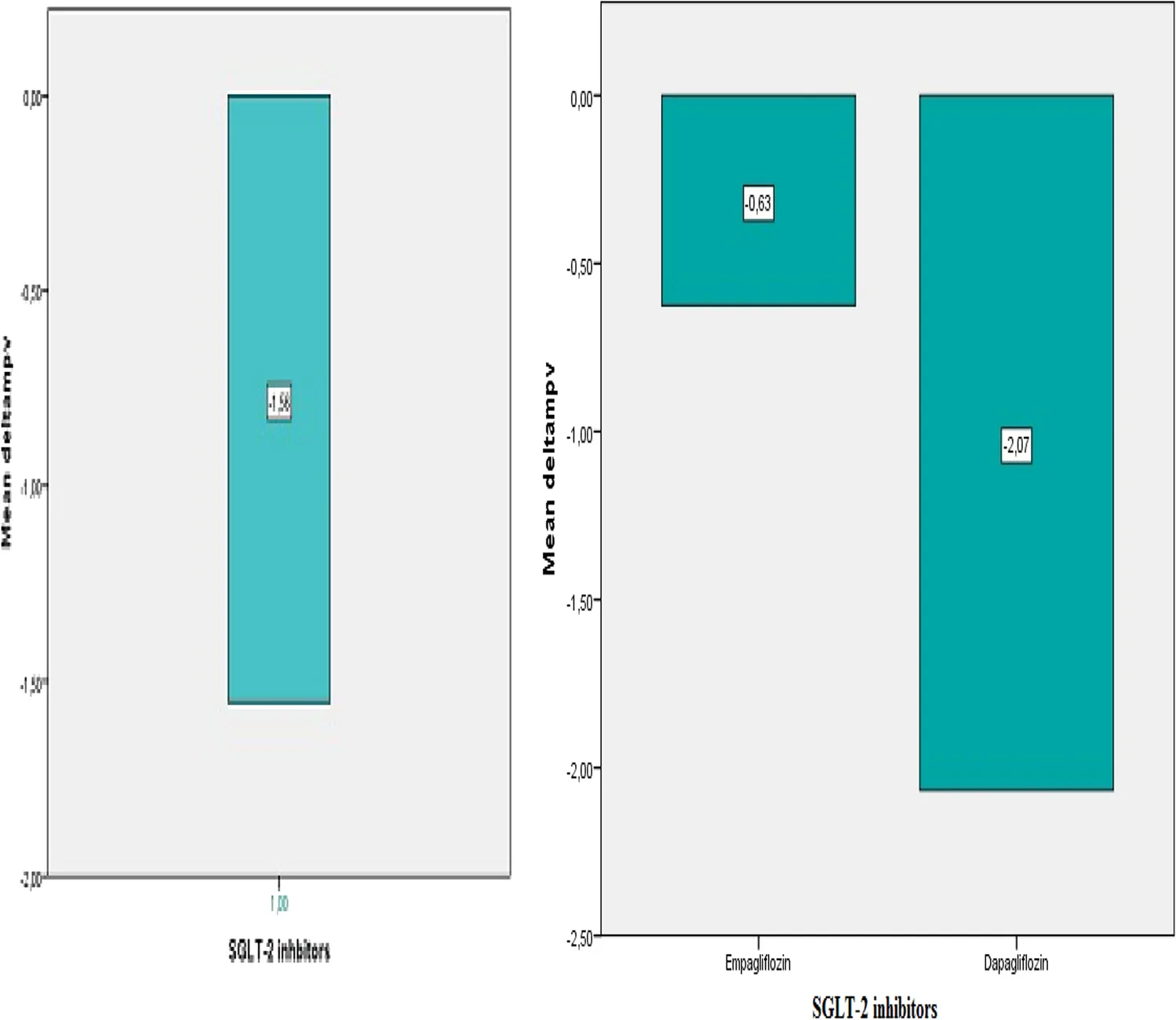
MPV changes in patients using SGLT-2 inhibitors

Changes in ΔMPV according to SGLT2 inhibitor subtypes are shown in Fig. 1.

A more significant decrease in MPV levels was observed in the dapagliflozin group compared to the empagliflozin group (*p* = 0.003).

No significant changes were observed in urea, creatinine, triglycerides, LDL-C, MCV, troponin, ProBNP, or hs-CRP levels following SGLT2 inhibitor treatment.

## Discussion

This study aimed to investigate the effects of SGLT2 inhibitors on hematological parameters in patients diagnosed with heart failure with reduced ejection fraction (HFrEF). In the present study, SGLT2 inhibitor therapy was associated with a significant reduction in MPV levels in patients with HFrEF, representing the primary finding of our analysis. In addition, platelet counts increased significantly, whereas hemoglobin and hematocrit levels showed a non-significant upward trend. Recent studies have demonstrated that SGLT2 inhibitors can regulate hematopoiesis through various cellular pathways and enhance erythropoiesis [6]. Consistent with the current literature, in our study, HGB and HCT levels increased at the 6-month follow-up after treatment initiation, although these changes were not statistically significant. Compared to placebo, SGLT2 inhibitor therapy may be associated with a slight increase in hematocrit. The diuretic effect of the drug could lead to hemoconcentration by causing volume depletion, which may underlie this increase [16].

Regarding the effects of SGLT2 inhibitors on platelet count, the literature remains inconsistent. In our study, a significant increase in PLT levels was observed in HFrEF patients. In diabetic populations, Eren et al. reported no increase in PLT levels, whereas Akhanlı et al. found a significant rise in PLT counts after SGLT2 inhibitor therapy [13, 15]. These discrepancies may be attributed to differences in study design, patient populations, and treatment durations. Nevertheless, independent of platelet count, a study by Kohlmorgen et al. demonstrated that dapagliflozin reduces CD62P (P-selectin) expression on collagen-activated platelets, decreases thrombin generation, lowers P2Y12 reaction units, attenuates platelet activation, and may reduce atherothrombotic risk [14]. Overall, SGLT2 inhibitors are thought to exert beneficial effects on platelet function and provide cardiovascular benefits. Platelets play a crucial role in hemostasis and thrombosis and are significantly involved in the management of active inflammation. During inflammation, large platelets release important molecules such as thromboxane A2, thromboxane B2, platelet factor 4, serotonin, and platelet-derived growth factor (PDGF) [3]. Therefore, upward changes in mean platelet volume (MPV) reflect increased platelet stimulation and/or production. Elevated MPV has been observed in atherosclerosis, acute coronary syndrome, acute ischemic stroke, obesity, dyslipidemia, and diabetes [17]. Given these findings, increased MPV has begun to be recognized as a cardiovascular risk factor due to its inclusion in routine blood tests, ease of use, and low cost.

All patients maintained stable antithrombotic regimens throughout the study period. Analysis revealed consistent MPV reductions across all antithrombotic therapy categories, including patients not receiving any antiplatelet therapy. This consistency suggests that the observed MPV decrease was independently driven by SGLT2i therapy rather than interactions with antithrombotic medications.Since antithrombotic therapy remained unchanged throughout the follow-up period, the observed changes in MPV are unlikely to be explained by alterations in antiplatelet or anticoagulant treatment. However, given the observational design of the study, a potential residual confounding effect cannot be completely excluded.

Another important finding of our study was the decrease in MPV levels. In our study, in the subgroup analysis, we observed lower MPV levels in the Dapagliflozin group compared to the empagliflozin group at 6 months. To our knowledge, there is no similar study on this subject in the literature. Dapagliflozin has been found to reduce 24-h blood pressure fluctuations because its sodium diuresis and sodium excretion effects last longer and are more stable than empagliflozin. Therefore, it is associated with a lower risk of developing cardiovascular diseases [18]. The more pronounced MPV reduction observed with dapagliflozin should be interpreted cautiously and may reflect pharmacodynamic differences rather than definitive superiority. In one study, it was determined that PLT volume increased after epinephrine infusion [3, 19]. The observed reduction in MPV may be related to the effects of SGLT2 inhibitors on neurohormonal pathways, including attenuation of sympathetic activity and modulation of the renin–angiotensin–aldosterone system.

SGLT2 inhibitors exert pleiotropic effects and offer cardioprotective and renoprotective benefits in patients with type 2 diabetes mellitus. The observed benefits in major adverse cardiovascular events (MACE), such as myocardial infarction and cerebrovascular events, may be partly related to the reduction in MPV, which has been consistently identified as an independent marker of cardiovascular risk in multiple studies [8–10]. In our study, a significant decrease in MPV was observed following SGLT2 inhibitor therapy. This reduction in MPV may be potentially linked to the positive effects of SGLT2 inhibitors on mortality.

In all patients included in our study, the maximal tolerable drug therapy was administered, and SGLT2 inhibitors were added subsequently. The majority of our patients had hypertension and diabetes, with a mean age of 64.6 years. To our knowledge, no previous studies have investigated the effects of SGLT2 inhibitors on MPV levels specifically in HFrEF patients. The only prior study examining SGLT2 inhibitors and MPV levels, conducted by Akhanlı et al., was limited to diabetic patients and excluded those with heart failure [15].

Platelet hyperreactivity is a well-known contributor to a prothrombotic state, resulting in increased coagulation, impaired fibrinolysis, and endothelial dysfunction. These hyperreactive platelets play a critical role in the pathophysiology of thrombotic events associated with poor prognosis in ischemic-origin heart failure [20]. A large study involving 3250 individuals found that those with elevated MPV had lower left ventricular ejection fraction and an increased risk of heart failure progression. Notably, individuals with MPV above the 75th percentile exhibited a 47% higher risk of heart failure deterioration. Additionally, in a cohort of 400 patients with chronic heart failure, MPV levels were shown to increase according to NYHA classification and to correlate with disease severity, suggesting that MPV may serve as a marker reflecting heart failure progression [21]. These findings suggest that one of the beneficial effects of SGLT2 inhibitors on heart failure might be mediated through reductions in MPV. However, no studies have compared platelet activity levels before and after SGLT2 inhibitor therapy specifically in HFrEF patients.

The dissociation between the increase in platelet count and the concomitant decrease in mean platelet volume (MPV) observed in our study warrants careful interpretation. Although hemoconcentration may partly influence platelet indices, it does not fully account for the concomitant reduction in MPV observed in our cohort. In contrast, our findings demonstrated a significant increase in platelet count accompanied by a significant reduction in MPV, with no meaningful correlation between changes in hematocrit and platelet count, and only a non-significant elevation in hematocrit. Collectively, this pattern supports the presence of a genuine bone marrow-mediated response characterized by enhanced production of smaller, less activated platelets rather than a simple effect of volume depletion. Such a mechanism may partly explain the reduced thrombotic risk reported in large-scale SGLT2 inhibitor trials.

Therefore, our findings provide useful insights into the effects of SGLT2 inhibitors on MPV in patients with HFrEF. Nevertheless, it should be noted that MPV can be influenced by various factors and is not a specific biomarker; thus, MPV values should be interpreted alongside clinical assessment and other diagnostic methods.

This study has several limitations. First, its retrospective and non-randomized observational design limits the ability to establish causality. Second, most patients were on aspirin or clopidogrel, which can affect MPV; however, since medication dosages remained unchanged during follow-up, the observed MPV changes were attributed to SGLT2 inhibitor therapy. Third, environmental factors such as climate and altitude differences, which might affect MPV, were not investigated in this study [21]. Finally, MPV was used as the sole indicator of platelet function; assessment of aggregation and resistance factors would have strengthened the validity of the results. Although the observed changes in MPV reached statistical significance, the magnitude of this reduction was relatively modest, and its clinical significance remains uncertain. Therefore, these findings should be interpreted with caution.

## Limitations

In addition, certain potential confounders that may influence MPV, such as smoking status, body weight changes, and blood pressure variations during follow-up, were not consistently available due to the retrospective design and could not be included in the analysis. Due to the relatively small sample size, multivariate analysis was not performed, as it may not provide reliable estimates.

## Conclusion

In conclusion, this retrospective study suggests that SGLT2 inhibitor therapy may reduce MPV levels and increase platelet counts in patients with HFrEF. Prospective, randomized, multicenter studies are warranted to confirm these findings.

## Data Availability

The datasets generated during and/or analyzed during the current study are available from the corresponding author on reasonable request.
